# First Evidence of Realized Selection Response on Fillet Yield in Rainbow Trout *Oncorhynchus mykiss*, Using Sib Selection or Based on Correlated Ultrasound Measurements

**DOI:** 10.3389/fgene.2019.01225

**Published:** 2019-12-20

**Authors:** Marc Vandeputte, Jérôme Bugeon, Anastasia Bestin, Alexandre Desgranges, Jean-Michel Allamellou, Anne-Sophie Tyran, François Allal, Mathilde Dupont-Nivet, Pierrick Haffray

**Affiliations:** ^1^ GABI, INRA, AgroParisTech, Université Paris-Saclay, Jouy-en-Josas, France; ^2^ MARBEC, Université de Montpellier, Ifremer, CNRS, IRD, Palavas-les-Flots, France; ^3^ LPGP, INRA, Rennes, France; ^4^ SYSAAF, LPGP-INRA, Rennes, France; ^5^ Les Aquaculteurs Bretons, Plouigneau, France; ^6^ LABOGENA-DNA, Jouy-en-Josas, France

**Keywords:** aquaculture, fillet yield, selective breeding, selection response, production efficiency, heritability

## Abstract

Fillet yield, the proportion of edible fillet relative to body weight, is a major trait to improve in fish sold processed, as it has a direct impact on profitability and can simultaneously decrease the environmental impact of producing a given amount of fillet. However, it is difficult to improve by selective breeding, because it cannot be measured on live breeding candidates, its phenotypic variation is low, and, as a ratio, it is not normally distributed and a same change in fillet yield can be the result of different changes in fillet weight and body weight. Residual headless gutted carcass weight (rHGCW) is heritable and highly genetically correlated to Fillet% in rainbow trout, and can be predicted by the ratio of abdominal wall thickness to depth of the peritoneal cavity (E8/E23), measured on live fish by ultrasound tomography. We selected broodstock based on rHGCW, measured on sibs of the selection candidates, on ultrasound measurements (E8/E23) measured on the selection candidates, or a combination of both. Seven broodstock groups were selected: fish with 15% highest (rHGCW+) or lowest (rHGCW−) EBV for rHGCW, with 15% highest (E8/E23+) or lowest (E8/E23−) EBV for E8/E23, with both rHGCW+ and E8/E23+ (Both+) or rHGCW− and E8/E23− (Both−), or with close to zero EBVs for both traits (Mid). Seven corresponding groups of offspring were produced and reared communally. At harvest size (1.5 kg mean weight), 1,561 trout were slaughtered, measured for the traits of interest, and pedigreed with DNA fingerprinting. Offspring from groups Both+, rHGCW+ and E8/E23+ had a higher EBV for rHGCW than the control group, while down-selected groups had a lower EBV. Looking at the phenotypic mean for Fillet% (correlated response), up-selected fish had more fillet than down-selected fish. The highest difference was between Both+ (69.36%) and Both− (68.20%), a 1.16% units difference in fillet percentage. The change in Fillet% was explained by an opposite change in Viscera%, while Head% remained stable. Selection using sib information on rHGCW was on average more efficient than selection using the candidates’ own E8/E23 phenotypes, and downward selection (decreasing Fillet%) was more efficient than upward selection.

## Introduction

Since modern selective breeding started in aquaculture in the 1970’s, growth rate has been and remains the main trait selected for in fish (Gjedrem et al., 2012). Higher growth rates increase production and shorten production cycles, but have little impact on production efficiency (and on the environmental impact of aquaculture), as the correlated response in feed efficiency is generally low to moderate (Knap and Kause, 2018). There is now growing interest in efficiency traits, that can increase production at a given level of inputs, or decrease inputs at a given production level. Among those traits, disease resistance is more and more included in selective breeding programs (Gjedrem, 2015). Feed conversion efficiency is also considered an important production efficiency trait, but remains difficult to improve by selective breeding in aquaculture, due to the inability to precisely record individual feed intake in the production environment, where fish are reared in large groups (de Verdal et al., 2018). Fillet yield, the ratio of edible fillet weight to body weight, has raised less interest. However, it can at the same time increase economic gain and decrease environmental impact, to produce a given quantity of edible fish meat in species sold as fillets. For a same amount of edible fillet available for human consumption, increasing fillet yield means less feed used, and less waste produced (Acosta Alba et al., 2015; Kankainen et al., 2016).

Selecting for increased fillet yield is a challenge in itself. First, fillet yield cannot be measured on live animals, which means that mass selection is not applicable. Possible selection methods are sib selection, where breeding values of the selection candidates are estimated based on measurements done on slaughtered sibs, or indirect selection, where traits measurable on live selection candidates and genetically correlated to fillet yield are used as indirect selection criteria. Second, fillet yield has low phenotypic variation (coefficient of variation in the 2%–6% range), and fillet weight is mostly proportional to body weight. This has led some authors to conclude that fillet weight was not possible to improve by selective breeding (Powell et al., 2008). To further complicate things, fillet yield is a ratio trait, and ratios have long been recognized as difficult traits to improve, because of their non-normality and because a given change in the ratio may be the result of different changes in its component traits. The expected genetic gain from truncation selection on ratio traits is difficult to compute using selection index theory, particularly when the component traits are correlated and have different means (Shirali et al, 2018). This is the case for the component traits of fillet yield, fillet weight, and body weight, which are very highly phenotypically and genetically correlated (*r*_P_ = 0.89–0.99, *r*_G_ = 0.93–0.99, see Fraslin et al., 2018 for a review). Despite these cumulated drawbacks, it has recently been shown by simulation based on real data that genetic gains in the range 0.30% to 0.95% units per generation should be achievable when selecting for improved fillet yield with 20% selection pressure in fish (Fraslin et al., 2018). Fillet yield may be used as a trait for selection, but residual fillet weight (rFW), which is fillet weight minus the expected fillet weight based on the linear regression of fillet weight on body weight, was proposed as a better alternative (Haffray et al., 2012a). This is because the heritability of ratio traits (here fillet yield = fillet weight/body weight) does not permit a reliable prediction of genetic change (Gunsett, 1987). On the contrary, the genetic change for residual fillet weight, which is not a ratio but reflects the variation in fillet yield to a large extent, can be reliably predicted from its heritability and phenotypic variance (Vandeputte, unpublished data).

Rainbow trout (*Oncorhynchus mykiss*) is the main fish species farmed in France, and one of the major salmonids species farmed worldwide (814.000 t in 2016, FAO). In France, production historically moved from a vast majority of pan-size (250–350 g) trout to mostly large (>1 kg) and very large (>2.5 kg) trout aimed at production of fillet, which are consumed fresh or smoked. Thus, fillet yield has become an increasingly interesting trait for French fish breeders. In this context, it was shown that fillet yield was heritable (*h*² = 0.31–0.44, Haffray et al., 2012a), confirming previous results from Norway (*h*² = 0.36–0.42, Gjerde and Schaeffer, 1989) and Finland (*h*² = 0.29, Kause et al., 2007). However, no reports of significant realized heritability are available for this trait. It was also shown that residual headless carcass weight was a suitable trait to include in the selection index when the aim is to increase fillet yield, because i) it is very highly genetically correlated with fillet yield (*r*_G_ = 0.98), ii) it has a higher heritability (*h*² = 0.55), and iii) it is easier to measure with less error, implying less technical skill than proper, repeatable filleting (Haffray et al., 2013). In addition, following on Bosworth et al. (2001) who showed that ultrasound tomography could be used to phenotypically predict processing yields in Channel catfish, ultrasound measurements were used as predictors of processing yields, especially in a mass selection context in rainbow trout French breeding programs (Haffray et al., 2004). More recently, Haffray et al. (2013) showed that the ratio of abdominal wall thickness (E8) to the depth of the peritoneal cavity (E23) was heritable (*h*² = 0.24) and genetically correlated to gutted carcass yield (*r*_G_ = 0.85) and to headless gutted carcass yield (*r*_G_ = 0.72). As this E8/E23 ratio can easily be measured on live candidates, it is an interesting trait to perform indirect individual selection for fillet yield, as headless gutted carcass yield is strongly genetically correlated to fillet yield, as outlined before.

In the present study, we performed divergent experimental selection for fillet yield in a rainbow trout population, comparing sib selection on residual headless carcass weight, indirect selection on E8/E23 measured on the candidates, and a combination of both. Parents were selected based on the two indices, and their offspring was evaluated for fillet yield and other morphological indices at 1.5 years of age, in order to evaluate realized selection response.

## Materials and Methods

### Production and Phenotyping of the Candidates and Sib-Test Populations

The traits measured and the rearing environments were indexed according to the ATOL ontology (Animal Trait Ontology for Livestock) and EOL (Environment Ontology for Livestock) available on ATOL (http://www.atol-ontology.com/index.php/en/les-ontologies-en/visualisation-en) website.

**Figure 1:**
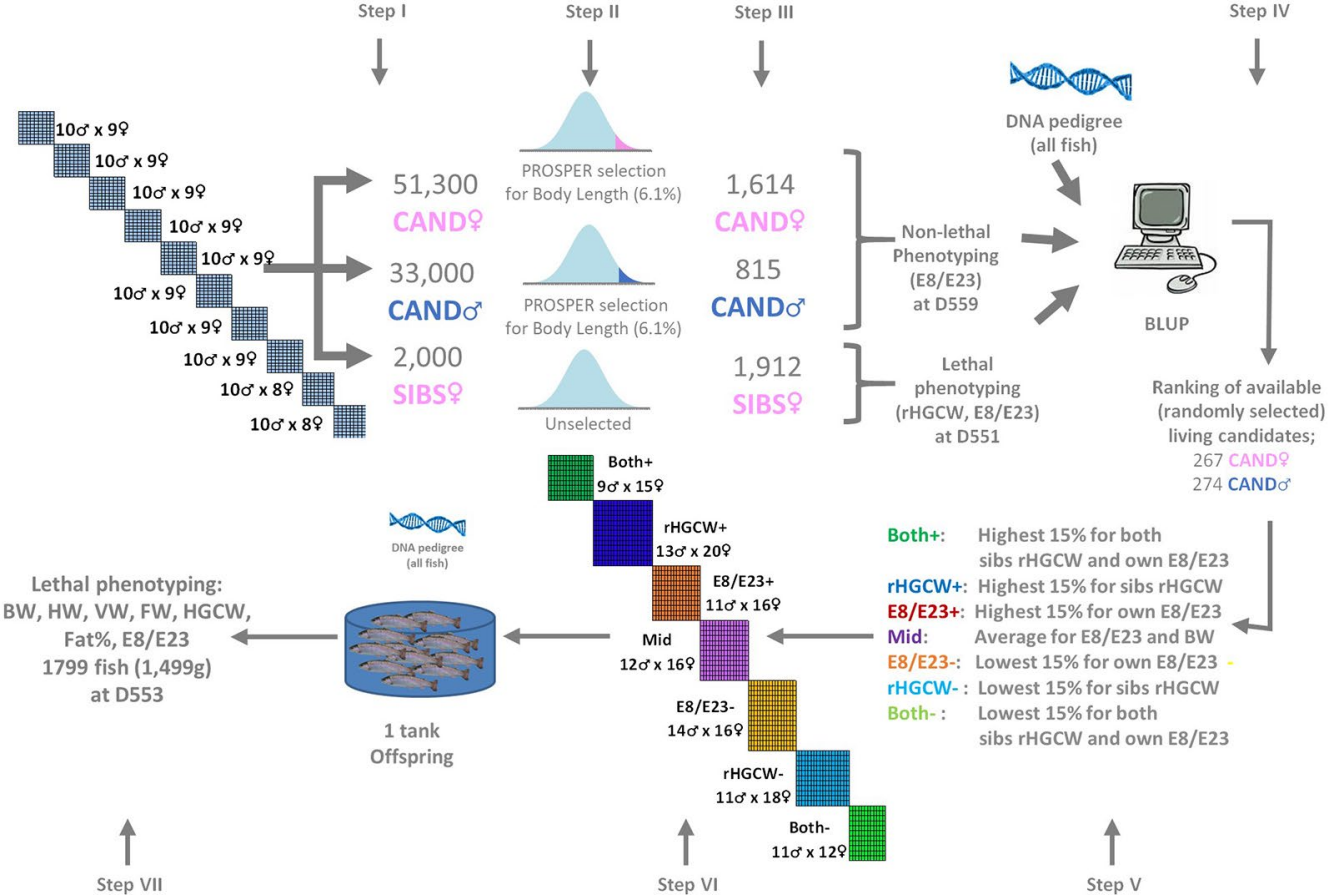
Summary of the experimental scheme. Selection candidates of each sex (CAND♀, CAND(neo)♂) are each reared as one mixed group of families from the initial 100 neomales x 88 females partial factorial design, and submitted to repeated phenotypic selection for body length (PROSPER, Chevassus et al., 2004) with a cumulated selection pressure of 6.1%. The SIBS groups is from the same families, reared without selection. Seven parent groups selected from the candidates (Both+, rHGCW+, E8/E23+, Mid, E8/E23−, rHGCW−, Both−) are used to produce seven offspring groups in the next generation, which are all reared in a single structure until final phenotyping. rHGCW, Residual headless gutted carcass weight; E8/E23, ratio of the abdominal wall thickness (E8) to the depth of the body cavity (E23) measured by ultrasound tomography; BW, body weight; HW, head weight; VW, viscera weight; FW, fillet weight, Fat%, Fillet fat percentage. The numbers of fish indicated at steps III and VII are the total numbers of fish phenotyped, the numbers of usable records (with pedigree and complete phenotype) are given in **Table 2**. Fish age is expressed in days post-fertilization (dpf).

The starting population of rainbow trout was taken from the commercial selective breeding program of Les Aquaculteurs Bretons (Plouigneau, France). The mating was performed in two days on 5–6 November 2013 following a partly factorial design, with 100 neomales (sex-reversed genetic females) and 88 females from the 5^th^ generation of the breeding programme, which was the base population for the one-generation experimental selection described in the present paper. The mating plan was composed of 10 independent full-factorial blocks of 10 neomales and 8 to 9 females, with an expected number of 880 full-sib families produced (**Figure 1**). Neomales (produced according to the European Council Directive 96/22/CE) were used because they are classically used by trout breeders to produce all-female offspring, which mature one year later than males and are thus better fit for large trout production.

A piece of fin from each parent was kept in 99% ethanol for further DNA extraction. Eggs from maternal half-sib families were incubated separately by dam. At the eyed stage, non-genetic maternal effects were managed by creating 10 new batches, each grouping spawns from eight dams with similar mean egg size (Haffray et al., 2012b). Each batch was then expected to contain 8 × 10 = 80 distinct full-sib families. Each batch was reared separately until 205 days post fertilization (D205) when they reached the same mean weight (BW, ATOL:0000351) of 10.4 ± 1.0g. At this age, and before any selection, 200 individuals per batch (n = 2000 in total) were sampled at random and pooled (i.e. from 880 expected families) to create the unselected slaughtered sibs (SIBS) group (Step I, see **Figure 1**). At the same time, 51,300 candidates from the same population were also pooled in one group at the Milin Nevez breeding center to create the future female selection candidates (CAND♀) group (Step I). These fish were then mass selected on growth (Step II) according to the within group PROSPER selection procedure (Chevassus et al., 2004), using body length (BL) at a given age as an indicator trait for growth rate. At D559, 1,614 candidates pre-selected with a cumulated selection pressure of 6.1% for BL were randomly sampled from the batch of candidates (Step III) and made available for further phenotyping.

The group of future neomale selection candidates (CAND♂) was created by sampling an equal number of eggs from each of the 10 initial egg pools. These fish were treated by feed supplemented with 3 mg/kg methyltestosterone at first feeding and during 600°C.days under veterinarian prescription, to induce the reversion of phenotypic sex to males. This was done under veterinary control, following EU Directive 96/22/EC. Each batch was reared separately until D205 when they reached the same mean weight (BW, ATOL:0000351) of 10g ± 1g. At that time, they were pooled in a batch of 33,000 CAND♂ neomales (Step I). These fish were mass selected on growth (step II) according to the within group PROSPER selection procedure (Chevassus et al., 2004) using BL at a given age as an indicator trait for growth rate. At D559, 740 candidates pre-selected with a cumulated selection pressure of 6.1% for BL were randomly sampled from the batch of remaining candidates (Step III) and made available for further phenotyping.

Water temperature varied from 3 to 20°C during the year. Tanks and raceways were supplied with the “first” water just entering in the fish farm. Fish were vaccinated against *Yersinia ruckeri* at D180. They were grown under non limiting oxygen availability (> 80% oxygen saturation, EOL:0000186) and fed to satiation using extruded commercial feed Neo Extra (Le Gouessant, Lamballe, France; 43% protein and 23% lipids) from 40 to 300 g and Neo Ultra (41% protein and 26% lipids; 25 ppm astaxanthine) from 300 g to the final harvesting. Density (EOL:0000043) increased with growth, but was kept to a maximum of 70 kg/m^3^.

The CAND♂ and CAND♀ groups were phenotyped on 18–19 May 2015 (D559, Step III). The fish were concentrated in the raceways by grid separators, then netted and anesthesized using Tricaïne (0.08g/l). They were tagged with ISO RFID glass tags (Biolog-ID, Bernay, France), and a piece of fin was collected from each fish for further DNA analysis. Each fish was weighed to the nearest 0.5 g (BW, ATOL:0000351), and the abdominal wall thickness (E8) and the depth of the peritoneal cavity (E23) were recorded by ultrasound tomography (Hospimedi LC100, 7.5 MHz) as described in Haffray et al. (2013). All fish were returned alive to their raceways.

The SIBS group was phenotyped from 8 to 12 May 2015 (D551 on average, Step III). Every day, approximately 400 fish were netted and immediately slaughtered by electronarcosis. A piece of fin was collected on each fish and stored in 99% ethanol for further DNA analysis. Each fish was weighed to the nearest 0.5 g (BW in g, ATOL:0000351). We also recorded headless gutted carcass weight (HGCW, ATOL:0002260) and untrimmed, skin-on, ribs-on fillet weight (FW, ATOL:0002304). Fillet fat (Fat%, ATOL:0001663) was recorded with a Distell Fish-FatMeter, following Douirin et al. (1998). Abdominal wall thickness (E8) and the depth of the peritoneal cavity (E23) were recorded by ultrasound tomography (Hospimedi LC100, 7.5 MHz).

### Selection of Parents for the Selection Experiment

Fish from the CAND♂, CAND♀ and SIBS groups were assigned to their parents using thirteen microsatellites by Labogena-DNA (ISO 17025 accredited, Jouy en Josas, France). This was done using the Accurassign software with a maximum-likelihood procedure (Boichard et al., 2014). The unique assignment rate was 89.5% in CAND♀, 89.9% in CAND♂ and 89.6% in SIBS. While a total of 2,140 fish (717 CAND♂, 1,423 CAND♀) had phenotypes for all traits studied and were uniquely assigned to their parents (so could be used in the calculations), only a random subsample of 541 fish (267 CAND♂, 274 CAND♀) were kept alive as selection candidates for the present experiment (Step IV).

Two traits were considered to perform experimental divergent selection for fillet yield. The first trait was residual headless gutted carcass weight (rHGCW, defined as HGCW minus the expected HGCW estimated from a linear regression of HGCW on BW), which was previously shown to be an easy to record surrogate for fillet yield (Haffray et al., 2012a). The second trait was the ratio of abdominal wall thickness to depth of the peritoneal cavity (E8/E23) which was shown to be the best simple predictor of fillet yield measurable on live rainbow trout (Haffray et al., 2013).

Prior to evaluating breeding values, the genetic parameters (heritabilities, correlations) of rHGCW, residual fillet weight (rFW), body weight, fillet Fat, and E8/E23 were estimated in the SIBS group (N = 1,694) with a multi-trait animal model, in order to be able to estimate the genetic correlations between the trait of interest (rFW) and the indirect traits (rHGCW, E8/E23) targeted for selection. Genetic correlations of HGCW and FW with BW were estimated with a bivariate animal model.

Then, breeding values were estimated using single trait models for each of the indirect traits, considering that, in the operational context of selective breeding, only the indirect traits would be routinely measured.

In order to perform indirect sib selection on rHGCW, the estimated breeding values (EBVs) of rHGCW were estimated as the solutions fitted by VCE 6.0 (Groeneveld et al., 2008) for the animal effect of the single trait model:

$$Y_{i}=\mu +{\text{animal}}_{j}+\varepsilon_{i}$$

Where Y_i_ is the rHGCW phenotype for an individual *i*, animal is the additive genetic effect of animal *j* and *ε* *_i_* is a random residual. Phenotypes for rHGCW were only available for the SIBS group, which were all females, and thus no fixed effects (group or sex) were needed in the model. The EBVs (animal effect) of CAND♂ and CAND♀ for rHGCW were estimated using only information from their slaughtered sibs and from the pedigree. Each candidate had on average 2.5 full sibs (0–15), 24.6 maternal half sibs (1–66) and 17.3 paternal half sibs (1–40).

In order to perform selection on E8/E23 (which can be measured on live selection candidates), the estimated breeding values (EBVs) of E8/E23 were estimated as the solutions fitted by VCE 6.0 (Groeneveld et al., 2008) for the animal effect of the single trait model:

$$Y_{ij}=\mu +{\text{group}}_{i}+{\text{animal}}_{j}+\varepsilon_{ij}$$

Where *Y*_ij_ is the E8/E23 phenotype for an individual *j*, group_i_ is the fixed effect of group (CAND♂, CAND♀), animal is the additive genetic effect of animal *j* and *ε* *_ij_* is a random residual. In this case, sex is confounded with the group effect, as CAND♂ are neomales and CAND♀ are females. Only phenotypes from the CAND♂ and CAND♀ groups were used, as it is likely that additional slaughtered sib information would not be available for selection in practice if the choice of indirect selection on E8/E23 was done.

Once sib-based EBVs for rHGCW and candidates based EBVs for E8/E23 were available for all CAND♂ and CAND♀ candidates, the following fish groups were identified (see **Figure 1**, Step V):

- The fish with the 15% highest and 15% lowest EBV for rHGCW (groups rHGCW+, rHGCW−).
- The fish with the 15% highest and 15% lowest EBV for E8/E23 (groups E8/E23+, E8/E23−).
- The fish which at the same time belonged to group rHGCW+ and E8/E23+ or to rHGCW− and E8/E23− (groups Both+, Both−).
- Fish with close to zero EBVs for both traits (group Mid).

Females and neomales from the different groups were manually assorted in order i) to identify at least 30 possible parents (15 neomales and 15 females) in each of the seven groups ii) to avoid as much as possible having neomales and females originating from the same full-sib family in a given group, to avoid the largest inbreeding effects. There was no overlap between the parents selected for the Both groups and the parents from the Echo and rHGCW groups.

This resulted in the selection, from the 541 fish with an EBV from the CAND groups in step IV (267 CAND♂ and 274 CAND♀), of the number of broodstock fish reported in **Table 1** for each selection group.

**Table 1:** Number of pre-selected broodstock (**Figure 1**, Step V) and of effectively used broodstock, which produced offspring in the next generation (in brackets, **Figure 1**, Step VI) for each of the selected parent groups.

Selection group	Number of CAND♀	Number of CAND♂	Total per group
rHGCW−	20 (18)	21 (11)	**41 (29)**
rHGCW+	21 (20)	21 (13)	**42 (33)**
Both+	15 (15)	15 (9)	**30 (24)**
Both−	14 (12)	14 (11)	**28 (23)**
E8/E23−	20 (16)	19 (13)	**39 (29)**
E8/E23+	20 (16)	19 (11)	**39 (27)**
Mid	16 (16)	17 (12)	**33 (28)**
**Total per sex**	**126 (113)**	**126 (81)**	**252 (194)**

### Production and Phenotyping of the Selected Offspring Groups

The mating was performed in one day on November 6, 2015 (Step VI, **Figure 1**). Within each of the seven selection groups (rHGCW−, rHGCW+, Both+, Both−, E8/E23−, E8/E23+, Mid), a full-factorial mating was performed with the neomales and females that produced sufficient amounts of gametes (see actual numbers into brackets in **Table 1**). Fertilized eggs from each of the seven groups were mixed in equal proportions after fertilization and incubated in a single incubator.

Water temperature varied from 3 to 20°C during the year. Tanks and raceways were supplied with the “first” water just entering in the fish farm. Fish were vaccinated against *Y. ruckeri* at D220. They were grown under non limiting oxygen availability (> 80% oxygen saturation, EOL:0000186) and fed to satiation using extruded commercial feed Neo Extra (Le Gouessant, Lamballe, France; 43% protein and 23% lipids) from 40 to 300 g and Neo Ultra (41% protein and 26% lipids; 25 ppm astaxanthine) from 300 g to the final harvesting. Density (EOL:0000043) increased with growth, but was kept to a maximum of 70 kg/m3.

**Figure 2:**
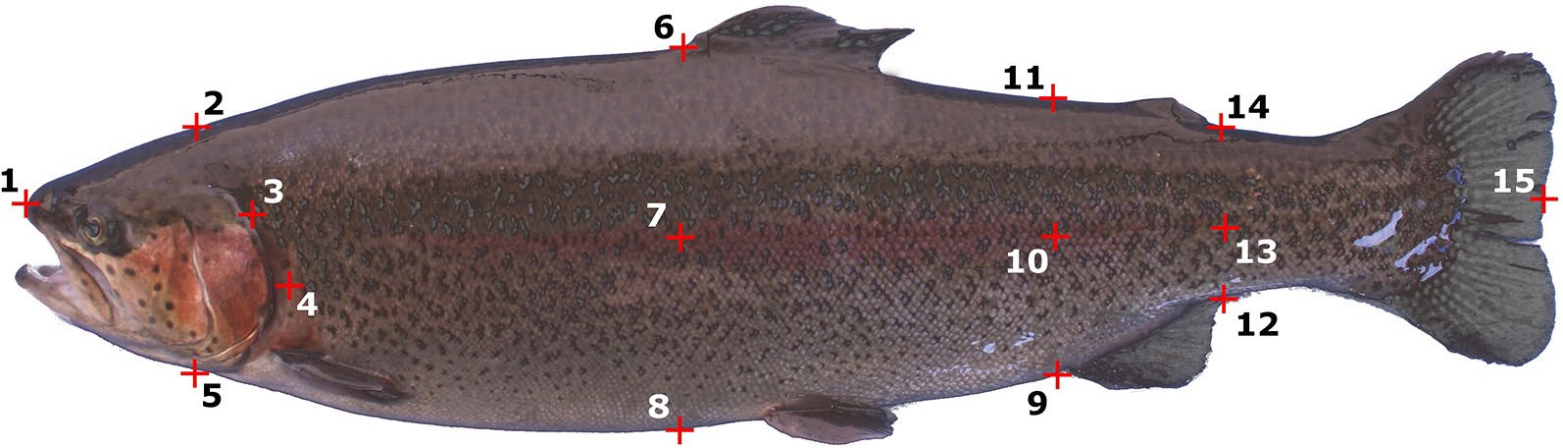
Localization of the 3D landmarks.

The offspring (N = 1,799, Step VII, **Figure 1**) were phenotyped from 15 to 19 May 2017 (D553 on average). Every day, approximately 400 fish were slaughtered by electronarcosis immediately after netting. A piece of fin was collected on each fish and stored in 99% ethanol for further DNA analysis. Each fish was weighed to the nearest 0.5 g (BW in g, ATOL:0000351), and the following weights were recorded: headless gutted carcass weight (HGCW, ATOL:0002260), untrimmed, skin-on, ribs-on fillet weight (FW, ATOL:0002304), head weight (HW, ATOL:0001545), and abdominal viscera weight (VW, ATOL:0002258). Abdominal wall thickness (E8) and the depth of the peritoneal cavity (E23) were recorded by ultrasound tomography (Hospimedi LC100, 7.5 MHz). Headless gutted carcass yield (HGC%, ATOL:0002261), fillet yield (Fil%, ATOL:0002305), head yield (Head%, ATOL:0001650), and Viscero-somatic index (Visc%, ATOL:0002259) were calculated as the ratio of the given compartment to body weight. The ratio of abdominal wall thickness to the depth of the peritoneal cavity (E8/E23) was also calculated. Fillet fat (Fat%, ATOL:0001663) was recorded as the mean of six measurements with a Distell Fish Fatmeter (three measurements on each side of the fish). Fifteen 3D landmarks (**Figure 2**) were digitized on each fish using a 3D digitizer (Microscribe G2LX).

### Analysis of Selection Response Data

The offspring were assigned to their parents using thirteen microsatellites by Labogena-DNA (ISO 17025 accredited, Jouy en Josas, France), in order to identify the selection group from which they were originated. This was done using the Accurassign software with a maximum-likelihood procedure (Boichard et al., 2014). The unique assignment rate was 89.2%.

The means of the different offspring groups were compared with a nested mixed model using proc MIXED in SAS (The SAS Institute):

$$Y_{\mathrm{ijkl}}=\mu +{\text{SG}}_{i}+{\text{sire}}_{j(i)}+{\text{dam}}_{k(i)}+\varepsilon_{\mathrm{ijkl}}$$

With *Y*_ijkl_ the phenotype of individual l, µ the overall mean, SG_i_ the fixed effect of selection group i, sire_j(i)_ the random effect of sire j nested within selection group i, dam_k(i)_ the random effect of dam k nested within selection group i and ε_ijkl_ the random residual. Due to the imbalanced design, approximate degrees of freedom for hypothesis testing were estimated with Satterthwaite’s method. Multiple comparison of means was performed with the Tukey-Kramer HSD test. This analysis was done on the target trait (Fil%) and on some key components of yield (Head%, Visc%), and also on BW and Fat% which are key production and quality traits.

For the traits selected, rHGCW and E8/E23, a multi-trait, multi generation model (including grandparents, CAND♂, CAND♀, SIBS, and offspring) was fitted with VCE 6.0, and the solutions were used as an estimate of the EBVs of the offspring. The model was run twice, first with only phenotypes from the parental generation (CAND♂, CAND♀ and SIBS = Parental EBV model) and secondly adding phenotypes from the offspring (Full EBV model). The parental EBV model estimates EBVs with the data available at the time of selection, and thus reflects the expected genetic divergence between offspring groups based on the performance of their parents. The full EBV model, including the phenotypes of the offspring, gives the best estimate of the actual EBVs of the offspring, and represents the genetic trend (selection response) for the traits studied. The effects of the seven offspring groups on the EBVs of the offspring generation, estimated from the parental and the full EBV models were analyzed using a nested mixed model with SAS-MIXED, as previously described. As the variance of EBVs depends on the quantity of information available, homogeneity of variance cannot be assumed, and thus no statistical tests were performed on group least square means.

The evolution of 3D morphology was assessed using the MorphoJ software (Klingenberg, 2011). Briefly, this method of geometric morphometrics consists of a Procrustes superimposition of landmarks. Then MorphoJ was used to perform a Linear Discriminant function analysis using the Procrustes coordinates of each fish (x, y, and z for each of the fifteen 3D landmarks, after Procrustes transformation) to examine the separation between two groups (rHGCW+ and rHGCW−, E8/E23+ and E8/E23−, and Both+ and Both−). The null hypothesis that the mean of the discriminant function is the same in both groups was tested with a T-square statistic in a permutation test with 1,000 random runs. The shape changes associated to the discriminant function were visualized with a wireframe graph. Leave-one-out crossvalidation was used to independently evaluate the reliability of the classification for each pair of groups tested.

## Results

### Genetic Parameters of Fillet Traits

The basic data of all fish used in the study are given in **Table 2**. Residual headless gutted carcass weight had a moderately high heritability (0.42, see **Table 3**), a little higher than that of residual fillet weight (0.38). The genetic correlations between them was high (0.88), confirming the good potential of rHGCW as a surrogate selection criterion for rFW. The indirect trait E8/E23 had a moderate heritability (0.21), and a moderately high genetic correlation with rFW (0.41). None of the above-mentioned fillet traits was significantly genetically correlated with body weight (*r*_G_ = −0.09 to 0.07), while HGCW and FW were very strongly genetically correlated to body weight (*r*_G_ = 0.995 and 0.991, respectively, data not shown).

**Table 2:** Basic data (mean ± standard deviation) of the different groups of fish used in the present study.

Group	CAND♂	CAND♀	SIBS	OFFSP
Gen.	G0	G0	G0	G1
Age (days)	559	559	551	553
N =	717	1,423	1,694	1,561
BW (g)	1,399 ± 101	1,491 ± 105	1,928 ± 365	1,499 ± 215
Fat (%)	9.8 ± 1.6	11.0 ± 1.9	12.8 ± 2.7	8.6 ± 1.5
E8/E23	0.128 ± 0.015	0.124 ± 0.016	0.108 ± 0.014	0.180 ± 0.029
Fillet (%)	NA	NA	68.3 ± 1.9	68.9 ± 1.8

CAND*♂,* neomale candidates to selection CAND*♀:* female candidates to selection. SIBS, sibs of CAND*♂* and CAND*♀* used to provide information on traits for which recording implies the sacrifice of the individual. OFFSP: average of the offspring groups used to evaluate selection response.

**Table 3:** Genetic parameters ( ± S.E.) of fillet-related traits estimated with a multi-trait animal model in the SIBS group of rainbow trout (N = 1,694).

	rHGCW	rFW	E8/E23	Fat	BW	BL
rHGCW	**0.42 ± 0.05**	0.88 ± 0.03	0.51 ± 0.09	0.23 ± 0.09	0.02 ± 0.09	0.09 ± 0.12
rFW	*0.80*	**0.38 ± 0.05**	0.41 ± 0.10	0.29 ± 0.09	0.07 ± 0.08	0.24 ± 0.11
E8/E23	*0.43*	*0.33*	**0.21 ± 0.04**	0.10 ± 0.12	-0.09 ± 0.07	0.00 ± 0.10
Fat	*0.15*	*0.06*	*0.08*	**0.41 ± 0.03**	0.25 ± 0.05	0.24 ± 0.10
BW	*0.19*	*0.15*	*0.03*	*0.33*	**0.31 ± 0.04**	0.84 ± 0.03
BL	*0.19*	*0.31*	*0.05*	*0.36*	*0.89*	**0.22** ± **0.03**

Heritabilities in bold on the diagonal, genetic correlations on the upper triangle, phenotypic correlations in italics on the lower triangle. rHGCW, residual headless carcass weight, rFW, residual fillet weight; E8/E23, ratio of the abdominal wall thickness to the depth of the peritoneal cavity; Fat, fillet fat (Distell fat-meter; BW, body weight.

### Selection Response

We first evaluated selection response on the traits selected, i.e. rHGCW and E8/E23 (**Table 4**), by studying the estimated breeding value of the seven offspring groups for those traits, provided by a multi-trait, multi-generation animal model.

**Table 4:** Comparison of the least square means ( ± S.E.) of the different offspring groups for estimated breeding values (EBVs) for the traits selected, i.e. residual headless carcass weight (rHGCW) and E8/E23.

	N =	rHGCW		E8/E23	
		Parent EBV	Full EBV	Parent EBV	Full EBV
Both+	131	15.25 ± 1.21	7.15 ± 2.11	0.0064 ± 0.0006	0.0034 ± 0.0021
rHGCW+	396	9.23 ± 0.66	5.49 ± 2.35	0.0013 ± 0.0004	0.0028 ± 0.0025
E8/E23+	123	4.28 ± 0.95	3.37 ± 1.74	0.0049 ± 0.0003	0.0039 ± 0.0020
Mid	163	−1.99 ± 0.90	−1.98 ± 2.61	−0.0010 ± 0.0003	−0.0041 ± 0.0016
E8/E23−	267	−5.23 ± 0.94	−5.15 ± 2.58	−0.0071 ± 0.0003	−0.0056 ± 0.0018
rHGCW−	208	−15.82 ± 1.20	−5.59 ± 2.35	−0.0022 ± 0.0004	0.0029 ± 0.0016
Both−	273	−17.28 ± 1.10	−16.36 ± 2.83	−0.0082 ± 0.0004	−0.0140 ± 0.0023
Sel+	650	9.21 ± 0.77	5.30 ± 1.29	0.0038 ± 0.0004	0.0035 ± 0.0014
Sel−	748	−12.10 ± 1.00	−8.53 ± 1.75	−0.0060 ± 0.0005	−0.0054 ± 0.0014

Sel+ represents the aggregation of all up-selected groups (Both+, rHGCW+, E8/E23+) and Sel− is the same for down-selected groups (Both−, rHGCW−, E8/E23−). EBVs were estimated based only on the phenotypes in the parental generation (parent) or adding phenotypes from the offspring generation (full).

For rHGCW, when only phenotypes from the parent generation were included (parent EBV), most offspring groups had largely different EBVs for the traits studied, consistent with the selection process. Average parent EBV for a group represents the expected genetic level of the offspring group, with the information available at the time of selection. The highest and lowest EBVs for rHGCW were observed for the Both+ and Both− groups, where sib information on rHGCW and individual information on E8/E23 were combined. The second most divergent groups for rHGCW were rHGCW+ and rHGCW−, whose parents were selected solely based on sib information for rHGCW. E8/E23+ and E8/E23− offspring groups also diverged for parental EBV of rHGCW, but to a lesser extent. As expected, the Mid group had a close to zero parental EBV for rHGCW. When phenotypic information from the offspring was included in the model, thus providing the real (and not only the expected) genetic level of the offspring groups, divergence was still apparent, though the groups were less separated The real genetic level was consistent with the expected one for groups E8/E23+, Mid, E8/E23− and Both−, but the genetic change was only half of the expectation for rHGCW+ and Both+, and one third of the expectation for rHGCW−.

In the case of E8/E23, offspring groups again had different EBVs for the traits studied when parent EBV was considered, again consistent with the selection process. Both+ and E8/E23+ had consistently high EBVs, and, symmetrically, Both− and E8/E23− had negative EBVs. Less difference was apparent for rHGCW+ and rHGCW−, for which the selection process did not use echography information. As before, the Mid group had a close to zero parental EBV. When looking at the real genetic level (including phenotypic information from the offspring in the model), there was mostly less divergence, and some unexpected results appeared. For rHGCW−, a positive EBV was observed, while the expectation was a mild negative EBV. For rHGCW+, there was also a positive EBV, but this time higher than the expectation. For Both+, the trend was positive but reduced compared to the expectation, similarly to what was observed with rHGCW. For Both−, there was a very strong negative trend, almost twice larger than the expectation. The Mid group had a mild negative genetic trend. Finally, the response for E8/E23+ and E8/E23−, for which parent selection was based solely on E8/E23, was consistent with the expectations, in direction and effect size.

The correlated response to selection was then studied on the target trait (Fillet%, **Table 5**) and correlated traits of interest. The differences observed between groups for fillet yield were small, but fillet yield in the Both+ and rHGCW+ groups was significantly higher (*P* < 0.05) than that in the Both− group. The largest difference was between the Both+ group and the Both− group (69.36% vs. 68.20%, a difference of 1.16%). When all selected groups were merged in Sel+ and Sel− (except Mid–Model 2 in **Table 5**), there was a significant difference (*P* = 0.0006) between the groups with 69.32% fillet in the up selected group vs. 68.65% in the down selected group.

**Table 5:** Comparison of the least square means ( ± S.E.) of the different offspring groups for yield traits (Fil%, Visc%, Head%), a quality trait (Fat%), and a production trait (body weight, BW).

Group		Traits measured in offspring				
	N =	Fil%	Visc%	Head%	Fat%	BW (g)
Both+	131	69.36 ± 0.27^a^	9.99 ± 0.21^b^	11.20 ± 0.14^a^	8.92 ± 0.29^a^	1,496 ± 29^ab^
rHGCW+	396	69.36 ± 0.19^a^	10.01 ± 0.15^b^	11.28 ± 0.10^a^	8.71 ± 0.21^a^	1,502 ± 20^ab^
E8/E23+	123	69.21 ± 0.26^ab^	9.97 ± 0.19^b^	11.48 ± 0.13^a^	8.66 ± 0.27^a^	1,440 ± 27^ab^
Mid	163	68.52 ± 0.24^ab^	10.23 ± 0.19^b^	11.60 ± 0.13^a^	8.17 ± 0.26^a^	1,413 ± 25^b^
E8/E23−	267	68.80 ± 0.21^ab^	10.66 ± 0.16^ab^	11.33 ± 0.11^a^	8.27 ± 0.23^a^	1,501 ± 21^ab^
rHGCW−	208	68.92 ± 0.24^ab^	10.37 ± 0.18^b^	11.39 ± 0.12^a^	8.78 ± 0.26^a^	1,535 ± 24^a^
Both−	273	68.20 ± 0.23^b^	11.34 ± 0.18^a^	11.30 ± 0.12^a^	8.69 ± 0.26^a^	1,537 ± 24^a^
Model 1 *F* (*P*-value)		*F*_6,90.4_ = 3.62 (*P* = 0.003)	*F*_6,110_ = 7.44 (*P* < 0.0001)	*F*_6,97.3_ = 1.10 (*P* = 0.37)	*F*_6,117_ = 1.15 (*P* = 0.34)	*F*_6,77.1_ = 3.38 (*P* = 0.005)
Sel+	650	69.32 ± 0.14^a^	9.99 ± 0.11^b^	11.32 ± 0.07^a^	8.75 ± 0.14^a^	1,484 ± 14^a^
Sel−	748	68.65 ± 0.13^b^	10.77 ± 0.11^a^	11.34 ± 0.07^a^	8.56 ± 0.14^a^	1,522 ± 13^a^
Model 2 *F* (*P*-value)		*F*_1,85.2_ = 12.69 (*P* = 0.0006)	*F*_1,98.8_ = 26.09 (*P* < 0.0001)	*F*_1,86.2_ = 0.04 (*P* = 0.84)	*F*_1,106_ = 0.92 (*P* = 0.34)	*F*_1,74.5_ = 3.94 (*P* = 0.051)

Sel+ represents the aggregation of all up-selected groups (Both+, rHGCW+, E8/E23+) and Sel− is the same for down-selected groups (Both−, rHGCW−, E8/E23−). Means with different subscripts in the same columns are different at P < 0.05 (Tukey-Kramer HSD adjustment).

The other yield traits Visc% and Head% showed that the gain in fillet yield was the consequence of a decreased viscero-somatic index, which was lower in Sel+ compared to Sel− although most of the unmerged selection groups were not significantly different. There was no reduction of the relative weight of the head in up-selected fish. There were also no differences in muscle fat content between the groups. There was a close to significant difference in body weight between the up- and down-selected groups (*P* = 0.051). Down-selected groups selected with indexes implying the use of rHGCW (Both−, rHGCW−) had a significantly higher BW than the Mid group, while Both+ and rHGCW+ were also heavier than Mid, although this was not significant (*P* > 0.10). These differences in BW led to the fact that the least square means for Fillet weight were not different among Sel+ and Sel− groups (1,045.6 ± 9.3 g in Sel−, 1,030.4 ± 9.8 g in Sel+, *F*_1,69_ = 1.34, *P* = 0.25), while least square means for Viscera weight were highly different between the same groups (148.0 ± 2.4 g in Sel+, 164.1 ± 2.3 g in Sel−, *F*_1,103_ = 24.02, *P* < 0.0001).

### 3D Morphology Differences Between Selected Offspring Groups

**Figure 3:**
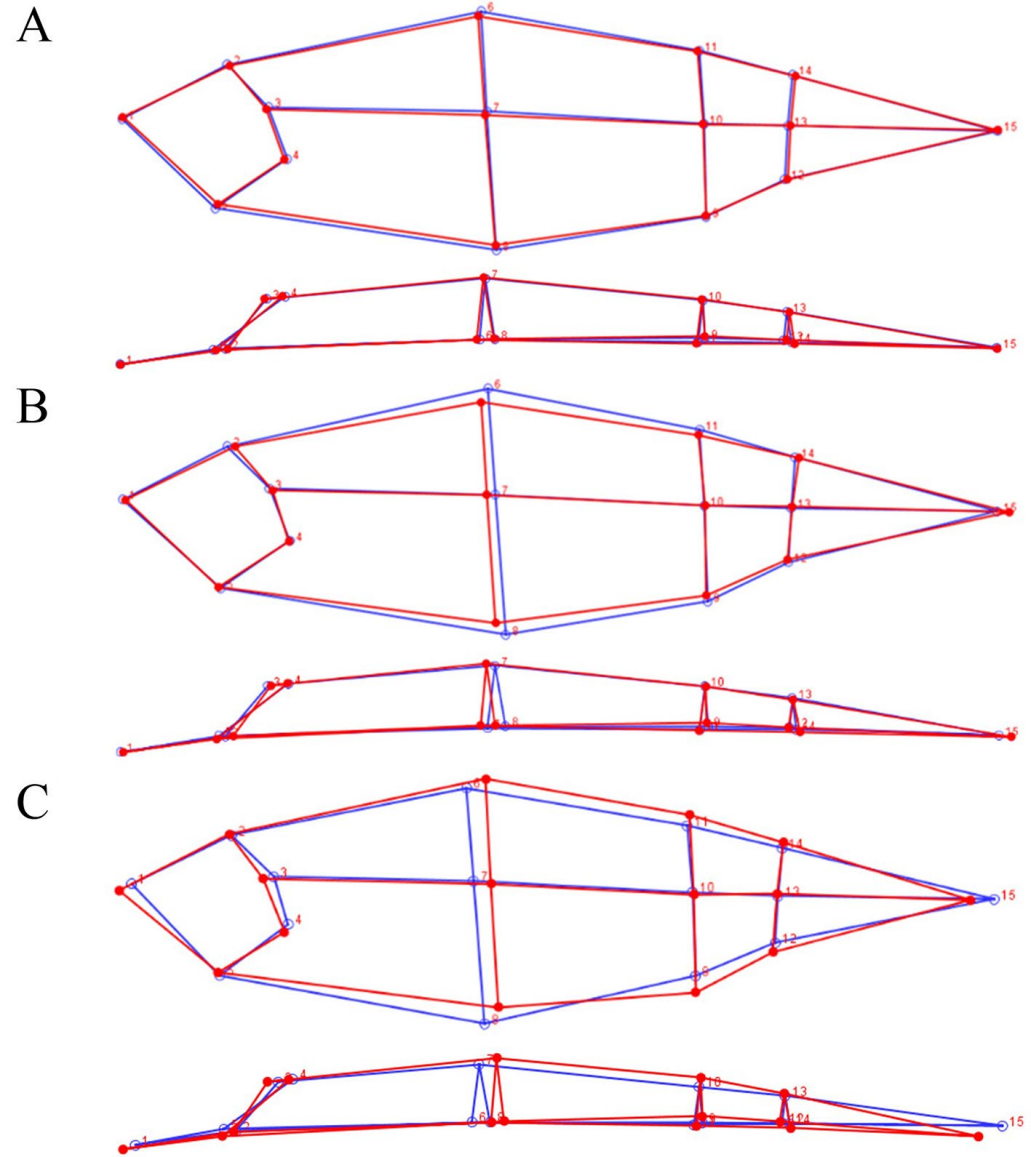
Lateral (top) and dorsal (bottom) view of the average 3D shape of the different offspring groups. **(A)** rHGCW+ (red) and rHGCW− (blue), **(B)** Echo + (red) and E8/E23− (blue), **(C)** Both+ (red) and Both− (blue).

**Figure 3A** illustrates the body shape associated to the discriminant function between the rHGCW+ and rHGCW− groups. The discriminant function was significant (p < 0.001), but only minor shape changes could be observed. The cross validation classification of the discriminant function was 64% positive rHGCW+ and 60% positive for rHGCW−, showing rather important shape overlapping between both groups.

In **Figure 3B**, the average body shape associated to the discriminant function between the E8/E23+ and the E8/E23− group is shown. The discriminant function was again significant (p < 0.001), and on the lateral view we can observe that E8/E23+ fish present a lower dorsal and ventral area especially around the dorsal fin. On the dorsal view, no major changes in body width were observed. The cross validation classification of the discriminant function was 67% concordant for E8/E23+ and 72% concordant for E8/E23−.

**Figure 3C** shows the differences in body shape between the Both+ and Both− groups. The discriminant function was significant (p < 0.001). On the lateral view, we can observe that Both+ fish present a higher dorsal area than Both− fish especially behind the dorsal fin. The Both+ fish also present a lower ventral area around the dorsal fin but a higher ventral area near the anus and in the caudal part. In dorsal view the Both+ fish present a higher body width behind the dorsal fin and in the caudal part. The head shape was also different between the two groups, with a longer snout but a shorter operculum for the Both+. The cross validation classification of the discriminant function was best for these two groups, 83% concordant for Both+ and 81% for Both−.

## Discussion

We showed for the first time in rainbow trout a significant and positive response to selection directed at improving fillet yield. There was a clear significant difference between the grouped up- and down-selected offspring (69.32% fillet vs. 68.65%, a 0.67% units difference in one generation). The response was even higher between the groups (Both+, Both−) whose parents were selected combining selection on the residual headless gutted carcass weight (rHGCW) of sibs and on their own phenotypes for the ultrasound ratio of abdominal wall thickness to depth of the peritoneal cavity (E8/E23). Between those groups, the divergence reached 1.16% units. To our knowledge, the only previous studies reporting selection response on fillet yield were performed on Nile tilapia. The first one gave a genetic gain in breeding value of 0.28% units over two generations (so on average 0.14% units per generation), using sib information to perform selection (Gjerde et al., 2012), and the second one an estimated gain of 0.2% units per generation over six generations with an index combining body weight and fillet yield (Thodesen et al., 2012). In our case, if we look at the difference in breeding value for rHGCW between the Both+ and the Mid group (**Table 4**), the gain in one generation is 9.13 grams, or 0.61% units headless gutted carcass. Thus, the first conclusion of the present study is that selection for improved fillet yield with significant impact is possible in rainbow trout.

This study is the first one to demonstrate the efficiency of indirect selection to increase fillet yield. The first trials to correlate fillet yield and morphology, either external morphology related to body shape (Cibert et al., 1999) or internal morphology (fillet thickness accessed with ultrasound imagery, Bosworth et al., 2001) took place quite a long time ago, and were followed by evaluations of the genetic correlations of such predictors with fillet yield (Rutten et al., 2005; Kause et al., 2007; Kocour et al., 2007; Haffray et al., 2013; Vandeputte et al., 2017). In these papers, the general conclusion was that several predictors (general shape analysis, head size, ultrasound measurements) were (genetically) correlated with fillet yield and could be used to improve it by indirect individual selection in a breeding program. However, no selection response data were available until the present study to demonstrate the practical applicability of such predictors. It has to be noted that in many cases, the predictors are complex linear combinations of external and/or internal morphological characteristics (Sang et al., 2009; Haffray et al., 2013; Vandeputte et al., 2017). This may raise issues concerning their application in batches or populations other than the one they have been established in. Luckily, for rainbow trout, the predictor with the highest genetic correlation to fillet yield was a simple one, the ratio of abdominal wall thickness to the depth of the peritoneal cavity (E8/E23). This probably was a key to success, and permitted an easy transfer to the breeding population used in the present study, which is not the same as the one on which the correlation was established. The heritability of E8/E23 in the present population was rather similar to the initial estimate (0.21 vs. 0.24 in Haffray et al., 2013) while the genetic correlation of E8/E23 with rHGCW was lower (0.51 vs. 0.72 in Haffray et al., 2013), but in the end it remained efficient.

Some unexpected phenotypic means were observed in some groups of fish in the present experiment (**Table 2**). First, the SIBS group, which is unselected, has a BW (1,928 g) which is much higher than the BW of any of the other groups (1,400–1,500 g), whereas age is similar. The difference with the OFFSP group can be explained by different annual temperature profiles, which have a very high impact on the growth of poikilothermic animals like fish. This is however not likely to explain the difference with the CAND groups, which are contemporary. In the fish farm, the SIB group was in a tank of its own. Due to the small number of fish, the rearing density of the SIBS group was much lower than that of the CAND groups, which favored growth for the SIBS. Moreover, in the selection for body length process, CAND groups were sorted several times, and each sorting was preceded by five days of fasting to ensure fish welfare during fishing and sorting (reducing oxygen consumption and excretion of feces in the water). This resulted in a lower mean weight in CAND groups compared to contemporary SIBS. A second point is that the average E8/E23 for the offspring (OFFSP) is much higher (0.180) than for the SIBS, CAND♂ and CAND♀ groups (0.108, 0.128, and 0.124 respectively). There is no clear explanation for this, the most likely is some operator variation, as this measurement is highly operator-dependent, and while it was the same operator for SIBS and CAND, it was a different one for OFFSP. Some environmental variation is also possible, as fish are very sensitive to temperature and feeding rate.

The gains observed here are a little lower than expected by simulation in another population of rainbow trout, where it was estimated that a 20% selection intensity on fillet yield should produce 0.6% to 0.7% units gain per generation (Fraslin et al., 2018). We could also see that the EBVs estimated with the full model (integrating the phenotypic data from the offspring) were in most groups lower than those estimated with only the data from the parental generation. When only phenotypic data from the parental generation are used, the mean “Parent EBV” of the offspring groups represents the expected genetic level of these groups, taking into account the EBVs of the parents known at the time of selection, combined with the observed offspring family structure (number of offspring produced by each parent), which then represents the expected genetic gain (**Table 4**). One important point here is that selection for the Echo and Both groups is based on biased EBVs, since the data used for selecting for E8/E23 are obtained only from the candidates, which were submitted before to phenotypic selection for the body length and thus have unknown population and family means. This is not optimal but represents the type of data a fish breeder would have if he decided that E8/E23 would be his criterion of choice, as in this case it would not be economically justifiable to rear and genotype an unselected sib group to phenotype it for E8/E23. However, as the genetic correlation between E8/E23 and body length is zero (**Table 3**), the expected bias is low. Selection for rHGCW is less likely to be biased as it is based only on sib information from the unselected SIBS group, thus family level EBVs are not expected to be biased. Still, candidates in the families are not a random sample of the family, as they have been submitted to phenotypic selection on BL. However, here again, the genetic correlation between BL and rHGCW is low (0.09 ± 0.12), hence the bias should be moderate. In any case, we see that the real selection response is lower than the expectation, a feature commonly seen in selection response experiments. Here, this could be linked to biases in the estimated genetic gain for rHGCW and E8/E23, as the EBVs estimated in the offspring with mixed model methodology depend on the estimates of heritability and genetic correlations and may differ from the real values (Thompson, 1986; Sorensen et al, 1994). Thus, the estimates produced here need to be taken with appropriate caution. In selection for ratio traits, the difference between expectations and realized gains may be very large. In a selection experiment to improve the egg mass to body weight ratio in the red flour beetle *Tribolium castaneum*, the observed selection response was 4 to 25 times lower than the expected one (Campo and Rodriguez, 1990). On another ratio trait, food conversion ratio, zero response was observed after six generations of selection in pigs (Webb and King, 1983). Still, this is not a generality, as a high realized heritability was achieved for selection on the body height to body length ratio in common carp (*h*²_r_ = 0.33–0.47; Ankorion et al., 1992). Using a similar approach as ours (selection on residuals), Egset et al. (2012) also managed to increase the ratio of caudal fin area to body area in the male guppy (*Poecilia reticulata*). In our case, selection was also performed on residuals, and was successful, although less than expected. It is unlikely that this success is only due to performing the selection on residuals, as it was shown that selection on residuals or on the ratio are equally effective in the case of fillet yield (Fraslin et al., 2018). We speculate that the success is partly due to the use of rHGCW as a selection trait, as this trait has a heritability similar to or better than that of fillet yield, and is much easier to record with minimal technical error.

This study also aimed at comparing sib selection with indirect selection using ultrasound measurements directly on selection candidates. Sib selection requires pedigree identification, as well as slaughtering of sib groups. Both requirements have significant costs, so indirect selection may be an interesting alternative to set up lower cost breeding programs. We saw little difference on fillet yield itself (**Table 5**) or on rHGCW (**Table 4**) between selection done with sibs (rHGCW groups) and individual ultrasound on candidates (E8/E23 groups). The response with E8/E23 was in general smaller than with rHGCW, but both methods provided gains. Here, more generations would be needed to establish if one method is more efficient than the other. In any case, the best method was the combination of the two, which led to the highest gains, with the same proportion selected. Even higher gains could be expected, as the combination of the two selection methods was performed by that choosing fish that independently passed the two thresholds, but were not optimally combined using selection index theory. Another possibility, suggested by Vandeputte and Haffray (2014) and applied in French breeding programs is to first cull candidates on their E8/E23 own phenotype by mass selection, without the cost of family information, and to apply sib selection on the pre-selected candidates only.

**Figure 4:**
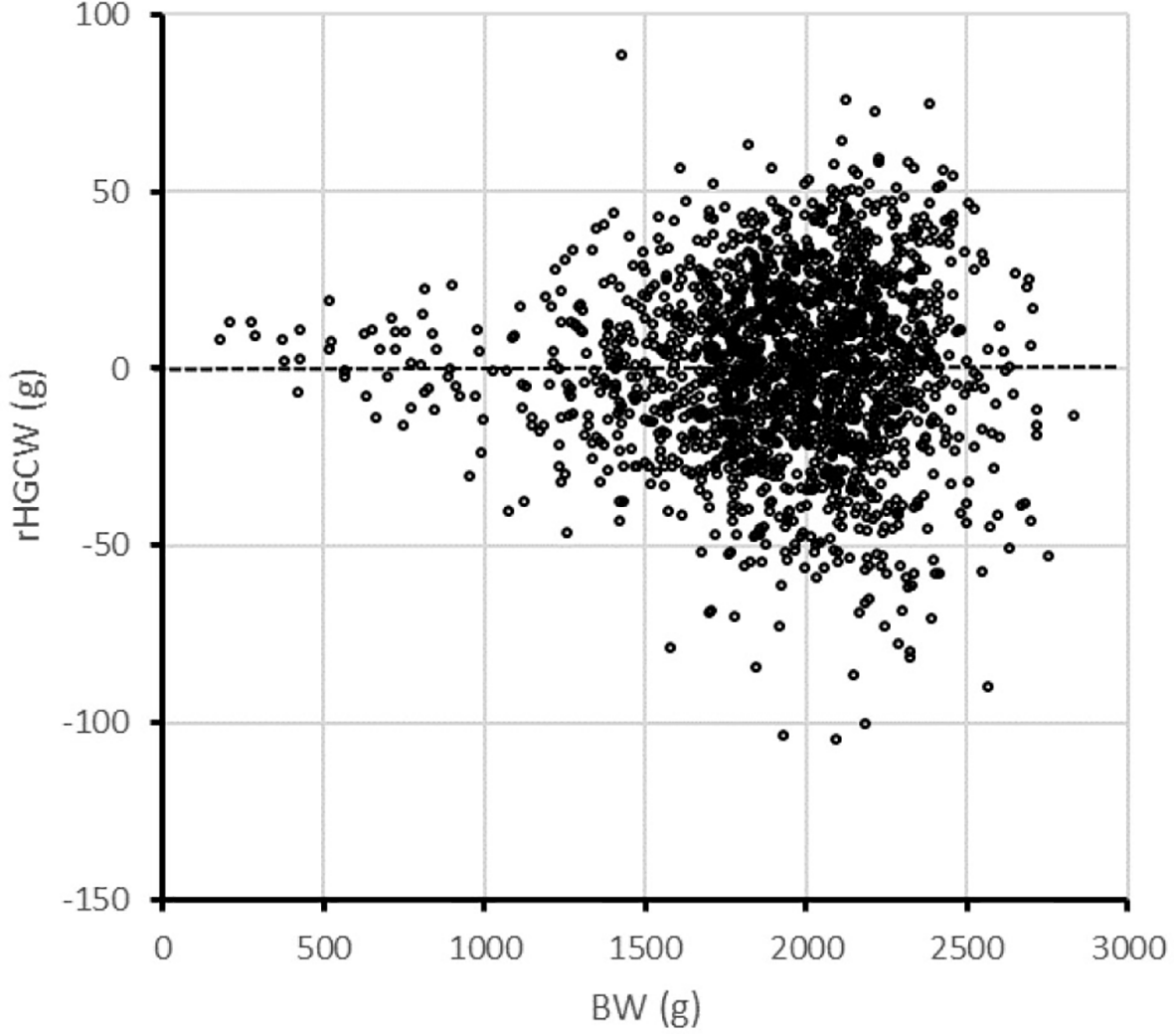
Distribution of residual headless carcass weight (rHGCW) as a function of body weight in the SIBS group of rainbow trout.

One surprising feature of the results is the “unselected” Mid group had a lower body weight than both the up- and down-selected groups. We could observe that rHGCW, although not genetically correlated with BW, increases in absolute values when BW increases (**Figure 4**), causing indirect positive selection for growth when rHGCW is selected, no matter it is selected downward of upward—and this is true although the genetic correlation of rHGCW with BW is close to zero (0.02 ± 0.09). When rHGCW is not selected, as in the Mid group, there is no such indirect selection, hence the Mid group has the smallest offspring. When rHGCW is only indirectly selected through E8/E23 in the E8/E23+ and E8/E23− groups, the effect on BW is more limited, as can be seen in **Table 5**. In this case, rather than using a simple residual, it may be better to use the log-residual of HGCW, which would suppress this scale effect, as was suggested in other cases (Egset et al., 2012; Vandeputte et al., 2017). Additionally, indirect selection for BW also caused a tendency for a higher weight in the offspring of down-selected parents (1,522 g in Sel− vs. 1,484 g in Sel+, *P* = 0.051). Fillet weight was similar in both offspring groups (1,045.6g in Sel+, 1,034.4g in Sel−, *P* = 0.25), but viscera weight was reduced by 16 g or 10% in Sel+ (148.0 g in Sel+, 164.1g in Sel−, *P* < 0.0001), showing Sel+ produced less waste than Sel− at the same body weight.

When studying the evolution of the different body parts in the seven offspring groups, it appeared that the main change was a decrease of the viscero-somatic ratio (Visc%) which was lower in the up selected groups (9.99% on average) compared to the down-selected groups (10.77%). This difference (0.78% units) is very close to the concurrent increase in fillet yield (0.67% units). Thus, in selected fish, viscera (which comprises mostly adipose tissue) development was replaced by a similar amount of muscle (fillet) development, which is what is expected to increase the value of the fish. The 3D morphology analysis confirms such differences in body parts development, for example Both+ fish present a lower abdominal part area, where the viscera are located, but a higher caudal part development leading to a higher muscle mass. This reduction of viscera is a general expectation of selection for fillet yield, as the genetic correlation of fillet yield and viscera yield is generally negative (*r*_G_ = −0.47 in rainbow trout, Kause et al., 2007, −0.25 in sea bass, Vandeputte et al., 2017).

Another quite generally expected impact of selection for fillet yield is a reduction of head size. Indeed, head size has even been suggested as an indirect criterion to improve fillet yield in carp (Kocour et al., 2007) and Nile tilapia (Rutten et al., 2005). In rainbow trout, it was also shown that there was a negative genetic correlation between the residuals of head and fillet weight (*r*_G_ = −0.53, Haffray et al., 2012a). In the present study, we did not see any correlated response in head size to selection for increased or decreased fillet yield. Still, there was a negative genetic correlation between rHGCW and residual head weight (*r*_G_ = −0.42 ± 0.08, data not shown), but at the same time a negative genetic correlation between residual viscera weight and residual head weight (*r*_G_ = −0.16 ± 0.08, data not shown) which may have somehow counteracted the expected negative effect of selection on rHGCW on head yield. It may also be that as head weighs much less than fillet, a proportionally similar decrease in weight may be more difficult to measure precisely enough to show a significant difference in just one generation.

The last important point is the asymmetry of selection response. When looking at the full EBV in **Table 4**, the contrast between the Mid group and the Both+ group (upward selection) is +9.13 g for rHGCW and +0.0075 for E8/E23, while the contrast between the Mid group and the Both− group is −14.38 g for rHGCW and −0.0099 for E8/E23. This may be indicative of the proximity of biological limits to selection. Indeed, it is trivial that 100% fillet yield cannot be achieved, and that there should be an upper limit to the proportion of fillet in a fish. Genetic variation for the traits is expected to remain even after numerous generations of selection if the size of the breeding populations is large enough (Hill and Bünger, 2003). However, some traits may reach biological limits such that moving beyond these limits produces drops in fitness that prevent continued changes. For example, in the Illinois corn selection experiment for protein content, the high line still increases over 25% protein after 100 generations, while the low line seems to have reached a plateau at approximately 5% protein (Moose et al., 2004). In a system closer to the question of fillet yield in fish, increasing breast yield in broiler chicken has led to increased respiratory capacity needs that cannot be met efficiently, causing pulmonary diseases (Wideman, 2001; Tickle et al., 2018). The biological limit for fillet yield is unknown in rainbow trout. In the present study, the average fillet yield of the ten highest performers (0.6% of the population) in the G1 generation, was 73.3%, to be compared to the mean of 68.9% for the same group. While we do not know if this representative of the biological limit, this may still give an idea of the range of progress that may be expected over generations. In this experiment, we did not observe a reduction of relative head size, as most of the gain was obtained by reducing the viscero-somatic index. It might be possible that by reducing relative head size, more gain could be obtained. However, as outlined by Haffray et al. (2012a), the respiratory organs of the fish (gills) are situated in the head, and it can thus be anticipated that reduction in head size would lead to negative fitness effects. Selection for increased fillet yield will then have to be combined with monitoring of the populations for the size of other body parts (including head, vertebral axis, fins, and scales), and for general robustness.

## Ethics Statement

This study was conducted in accordance with EU Directive 2010-63-EU on the protection of animals used for scientific purposes. The fish were reared according to normal husbandry practices in the breeding programme of Les Aquaculteurs Bretons, and were not subjected to practices likely to cause pain, suffering, distress or lasting harm equivalent to, or higher than, that caused by the introduction of a needle in accordance with good veterinary practice. As such, the experiment did not require approval by an Ethics Committee, in accordance with Article 2.5 of the Directive.

## Author Contributions

MV, PH, JB, MD-N, and AD contributed conception and design of the study. MV, JB, AB, AD, A-ST, and PH collected the data. AB and A-ST organized the database. MV, JB, AB, J-MA, FA, and A-ST performed data analysis. MV wrote the first draft of the manuscript. AD and JB wrote sections of the manuscript. All authors contributed to manuscript revision, read and approved the submitted version.

## Funding

This study was supported by the European Union’s Seventh Framework Programme (KBBE.2013.1.2-10) under grant agreement no. 613611 FISHBOOST (http://www.fishboost.eu/).

## Conflict of Interest

The authors declare that the research was conducted in the absence of any commercial or financial relationships that could be construed as a potential conflict of interest.
